# Angina bullosa haemorrhagica: A 14-year multi-institutional retrospective study from Brazil and literature review

**DOI:** 10.4317/medoral.24870

**Published:** 2021-09-25

**Authors:** John Lennon Silva-Cunha, Israel Leal Cavalcante, Caio César da Silva Barros, Fernanda Aragão Felix, Luan Borges Venturi, Larissa Santos Amaral Rolim, César Luis Porpino Santos da Silva-Júnior, Emanuel Mendes Sousa, Éricka Janine Dantas da Silveira, Michelle Agostini, Mário José Romanach, Oslei Paes de Almeida, Sílvia Ferreira de Sousa, Bruno Augusto Benevenuto de Andrade

**Affiliations:** 1 DDS, MSc, PhD student. Department of Oral Diagnosis, Piracicaba Dental School, University of Campinas (UNICAMP), Piracicaba, Brazil; 2 DDS, MSc, Professor. Department of Dentistry, University of Fortaleza (UNIFOR), Fortaleza, Brazil.; 3 DDS, MSc, PhD student. Department of Dentistry, Postgraduate Program in Dental Sciences, Federal University of Rio Grande do Norte (UFRN), Natal, Brazil; 4 DDS, MSc, PhD student. Department of Oral Surgery and Pathology, School of Dentistry, Universidade Federal de Minas Gerais, Belo Horizonte, MG, Brazil; 5 DDS. Hospital Municipal Dr. Cármino Caricchio, São Paulo, Brazil; 6 DDS. Multicampi Medical Science School, Multiprofessional Residency Program, Federal University of Rio Grande do Norte (UFRN), Caicó, Brazil; 7 DDS, MSc. Department of Oral Diagnosis and Pathology, School of Dentistry, Federal University of Rio de Janeiro (UFRJ), Rio de Janeiro, Brazil; 8 DDS, PhD, Professor. Department of Dentistry, Postgraduate Program in Dental Sciences, Federal University of Rio Grande do Norte (UFRN), Natal, Brazil; 9 DDS, PhD, Professor. Department of Oral Diagnosis and Pathology, School of Dentistry, Federal University of Rio de Janeiro (UFRJ), Rio de Janeiro, Brazil; 10 DDS, PhD, Professor. Department of Oral Diagnosis, Piracicaba Dental School, University of Campinas (UNICAMP), Piracicaba, Brazil; 11 DDS, PhD, Professor. Department of Oral Surgery and Pathology, School of Dentistry, Universidade Federal de Minas Gerais, Belo Horizonte, MG, Brazil

## Abstract

**Background:**

Angina bullosa haemorrhagica (ABH) is characterized by the recurrent appearance of blood blisters on the oral mucosa, mainly in adults' soft palate. In general, the blisters rupture spontaneously, lacking the necessity for biopsy. We report the clinical features of 23 ABH cases, emphasizing the clinical behavior and the management of these conditions.

**Material and Methods:**

A retrospective descriptive cross-sectional study was performed. A total of 12,727 clinical records of oral and maxillofacial lesions from four dental services in Brazil were analyzed. Clinical data were collected from the clinical records and evaluated.

**Results:**

The series comprised 12 males (52.2%) and 11 females (47.8%), with a mean age of 56.8 ± 14.6 years (ranging: 24-82 years) and a 1.1:1 male-to-female ratio. Most of the lesions affected the soft palate (n = 15, 65.2%). Clinically, the lesions presented mainly as an asymptomatic (n = 17, 73.9%) blood-filled blister that ruptured after a few minutes or hours, leaving an erosion. The masticatory trauma was the most frequent triggering event. No patient had coagulation disorders. A biopsy was performed in only four cases (17.4%). Treatment was symptomatic with a favorable outcome.

**Conclusions:**

ABH is still poorly documented in the literature, and its etiology remains uncertain. ABH mainly affects the soft palate of elderly adults and has a favorable evolution in a few days. The therapeutic approach is often focused only on the relief of symptoms. However, it can share some clinical features with more serious diseases. Therefore, clinicians must recognize these lesions to avoid misdiagnosis.

** Key words:**Angina bullosa haemorrhagica, diagnosis, oral mucosa blisters.

## Introduction

Angina bullosa haemorrhagica (ABH) was described in 1967 by Badham as an alteration that causes recurrent hemorrhagic blisters, which rupture easily located exclusively in the oropharyngeal or oral mucosa in sites particularly exposed to trauma (1). However, this condition can occur in any location of the oral cavity. Although several single case reports and small case series have been published in the literature (2-7), the etiology of ABH remains poorly understood, and lesions are often underdiagnosed (8).

The differential clinical diagnosis of ABH is broad and includes several vesiculobullous lesions and hematological diseases (8,9). After the blister rupture, differential diagnosis becomes broader and challenging and includes several disorders that cause post-bullous erosions (8). Although the disease's clinical history is an essential factor in the diagnosis of ABH, there is wide variation in the literature on how to obtain an accurate diagnosis (8).

Thus, this study aims to report a series of 23 new cases of oral ABH. To the best of our knowledge, this is the largest Brazilian series on ABH to date. Additionally, we provide a comprehensive literature review describing the clinical and therapeutic characteristics of ABH and discuss the proposed diagnostic criteria for this condition.

## Material and Methods

- Study design and data obtaining

In this retrospective cross-sectional study, a total of 12,727 clinical records of patients with oral and maxillofacial lesions were analyzed (2006-2020), and all cases diagnosed as ABH were recovered. The records were retrieved from the archives of four dental services: Department of Oral Diagnosis and Pathology, Federal University of Rio de Janeiro (UFRJ), Rio de Janeiro, Brazil; Department of Dentistry, Federal University of Rio Grande do Norte (UFRN), Natal, Brazil, and two private practice office.

Patients that met the diagnostic criteria for ABH proposed by Ordioni *et al*. (2019) were included (Table 1).

**Table 1:**
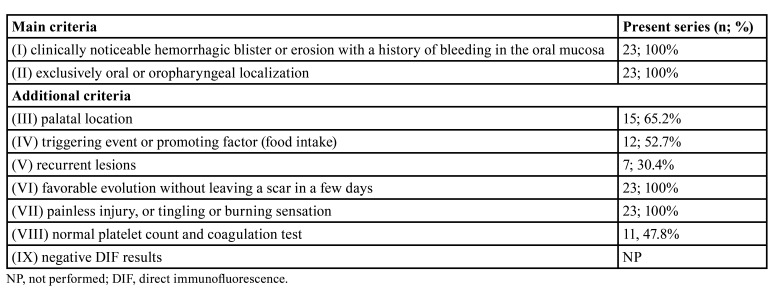
Diagnostic criteria for the diagnosis of angina bullosa haemorrhagica proposed by Ordioni *et al*. (2019).

Clinical data were collected from the clinical records and evaluated. Additional information on treatment and follow-up was obtained from the requesting dentists. The histological slides were recovered when a biopsy was performed, and the histopathological findings were evaluated.

- Analysis

Data were analyzed and described using the Statistical Package for the Social Sciences 22.0 (SPSS, IBM Corp., Armonk, NY, USA). Continuous variables were expressed as mean, median, and standard deviation (SD). Categorical variables were expressed as the absolute number of cases and percentage values.

## Results

In the present study, 23 cases were retrieved from the archives of four dental services between 2006 and 2020. The prevalence of ABH was 0.18% from a total of 12,727 diagnostics. The clinical data are summarized in Table 2.

**Table 2:**
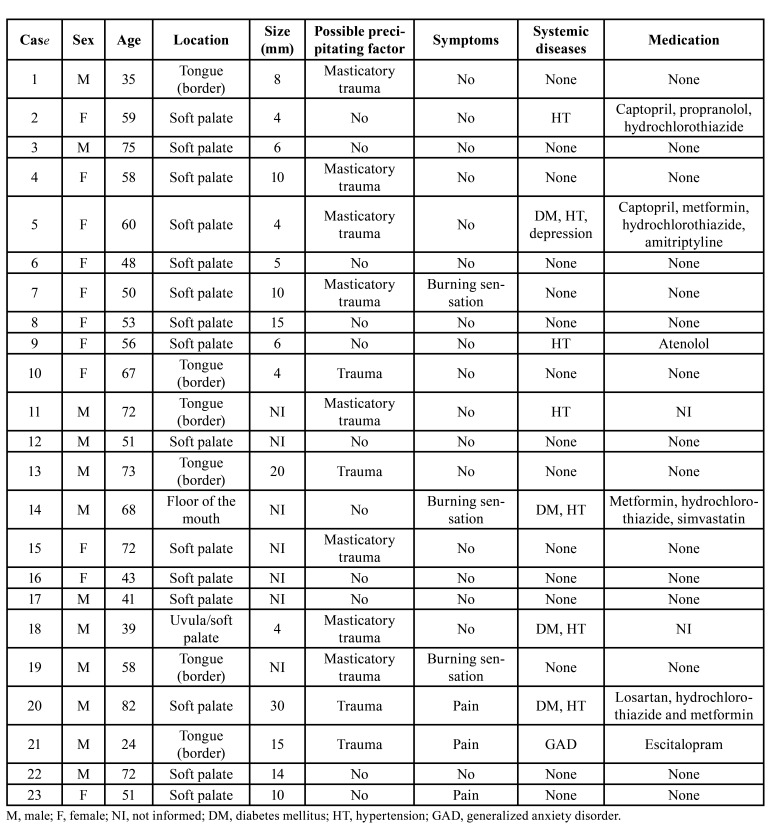
Clinical features of 23 patients with angina bullosa haemorrhagica of the present series.

ABH was slightly more prevalent in males (n = 12, 52.2%), with a 1.1:1 male-to-female ratio and a mean age of 56.8 ± 14.6 years (ranging: 24-82 years). Patients in the sixth (n = 8, 34.8%) and eighth (n = 5, 21.7%) decades of life were most affected. The soft palate was the main site (n = 15, 65.2%), followed by the tongue's lateral border (n = 6, 26.1%). Other less common locations included floor of the mouth (n = 1, 4.3%) and uvula (n = 1, 4.3%).

**Figure 1:**
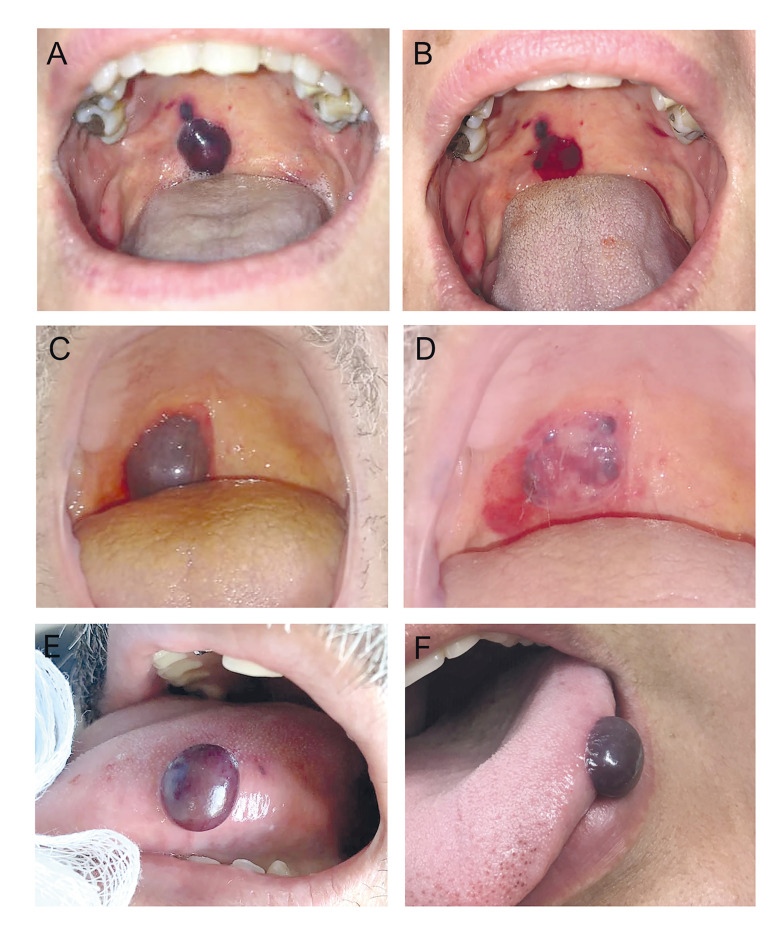
Clinical aspects of angina bullosa haemorrhagica. (A) Two blood-filled blisters in the right side of the soft palate and (B) clinical aspect immediately after the incision and drainage of the blood content. (C) Large blood blister on the soft palate, and (D) ruptured blister with epithelial remnant and blood clot (3 hours later). (E, F) Clinical aspects of lesions in the border of the tongue.

Clinically, most lesions showed a similar clinical appearance (Fig. 1). Patients described a blood-filled blister, whether or not preceded by a burning or tingling sensation. The blisters often had an equimotic halo and ruptured after a few minutes or hours, leaving an erosion, in most cases, asymptomatic (n = 17, 73.9%), which healed without scarring in a few days. In most cases, ABH appeared as a solitary lesion (n = 18, 82.6%), and less frequently as two (n = 3, 13.0%) or multiple (n = 1, 4.4%) blisters. The lesions' size varied from 0.4 to 3.0 cm, with a mean size of 1.0  ± 0.7 cm. There was no description of the Nikolsky sign in none of the cases. Blood-filled blisters were intact in 14 (60.9%) of the 23 cases. A small amount of blood was seen inside the ruptured lesion in 6 cases (26.1%).

There was no identifiable triggering event or promoting factor in 11 cases (47.8%). However, a previous history of a traumatic event was reported by 12 patients (52.2%). No patient reported a family history of ABH. Regarding medical records, most patients had no comorbidities (n = 15, 65.2%); however, four had hypertension and diabetes (17.4%), three only hypertension (13.0%), and one generalized anxiety disorder (4.4%). No patient had coagulopathies. Hematological exams did not show any changes when requested (n = 11, 47.8%). All cases fulfilled at least six of the nine diagnostic criteria proposed by Ordioni *et al*. (2019), including criteria I and II (Table 1).

**Figure 2:**
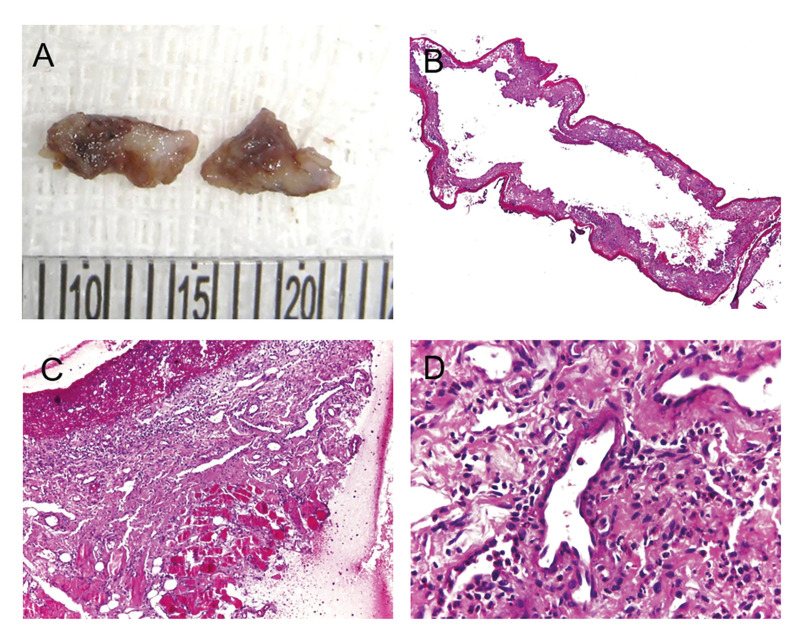
Macroscopic and histopathological aspects of angina bullosa haemorrhagica. (A) The gross specimens showed irregular shapes and cut surfaces with whitish and brownish areas. (B) Oral mucosa lining epithelium showing areas of detached connective tissue and hemorrhage. (C) Area of ulceration and granulation tissue and (D) diffuse mixed inflammatory infiltrate. (B-D, H, E stain; original magnifications: B, 40×; C, ×200; D, ×400).

In most cases (n = 15, 65.2%), no specific treatment was performed. However, analgesics and mouthwash with 0.02% chlorhexidine gluconate solution were prescribed in four cases (17.4%). In addition, only four cases (17.4%) were submitted to excisional biopsy. Microscopically, the findings were nonspecific and showed an area of ulceration, hemorrhage, and a diffuse mixed inflammatory infiltrate (Fig. 2). Seven patients (30.4%) reported episodes of local recurrence.

## Discussion

ABH is a benign disorder characterized by the acute onset of a blood-filled blister in the oropharyngeal or oral mucosa (1,5,8,10). Although it is often described as a rare condition, some authors suggest that ABH is probably underdiagnosed due to the clinicians' lack of knowledge and its self-limited nature and spontaneously favorable evolution (8,11). Thus, it is difficult to determine the true incidence and prevalence of this condition. However, in the present study, the sample represented about 0.18% of the total lesions diagnosed in the referred services, similar to a previous study conducted by Grinspan *et al*. (11).

ABH tends to occur in adults between the fifth and the seventh decades of life, with a mean age of 55.4 years (8), similar to the current series. Although some previous studies have shown a female predominance of 1.4:1 (5,8), the present study revealed a practically equal distribution with a 1.1:1 male-to-female ratio. Most of the lesions occur on the soft palate, followed by the tongue's lateral border (8), similar to the current series.

The etiology and pathogenesis of ABH remain unclear, and several hypotheses have been proposed (8). Some authors report an association between ABH and long-term use of inhaled corticosteroids (12). Chronic use of these drugs may impair collagen formation and cause atrophy of the epithelial tissue (13). Disorders of the collagen and elastic fibers of the oral mucosa result in less anchorage of blood vessels, which can cause hemorrhagic lesions after trauma (8,14), predisposing patients to these conditions. Some authors have also suggested a possible association of ABH with hypertension, diabetes mellitus, hyperglycemia, and a family history of diabetes (11,15). However, these associations were weak and appeared to be speculative (8). Curiously, unlike our results, a small percentage of patients (3.5%) reported similar cases in family members; however, it is still not possible to state a probable familial genetic tendency (8).

A previous history of trauma has been the main finding reported in most cases (> 80%), and it seems to be the most relevant etiological agent (5,8). Trauma associated with hard, hot, or spicy food intake was the most frequently reported factor (8), similar to the present series. The trauma leads to a loss of cohesion between the epithelium and connective tissue (8). A possible fragility of the vasculature and/or elastin and/or collagen in some patients could favor subepithelial hemorrhages (2,8,16,17). However, further studies are needed to confirm this hypothesis.

The differential diagnosis of ABH is broad and includes especially two groups of lesions, namely, (I) vesiculobullous lesions, such as pemphigus vulgaris, mucous membrane pemphigoid, bullous pemphigoid, epidermolysis bullosa acquisita, linear IgA dermatosis, amyloidosis, oral bullous lichen planus, and dermatitis herpetiformis, and (II) hematological diseases, such as von Willebrand's disease, thrombocytopenia, and leukemia (8,17).

Although the diagnosis of ABH can be based on clinical examination, history, and follow-up, there is significant variability in the diagnostic approach and management of the disease in the literature (8). In general, a clinically noticeable hemorrhagic blister or an erosion with a history of oral bleeding due to the rupture of a previous blister exclusively located in oral mucosa is essential for the diagnosis (8,18). Besides, the presence of a triggering event (chewing), isolated or recurrent lesions on the soft palate with favorable evolution within a few days without leaving scars also favors the diagnosis of ABH (8). On the other hand, other authors advocate a comprehensive review of the patient's systemic condition, especially in patients with suspected hemostasis abnormalities (3,19). In these cases, tests such as platelet counts and coagulation tests should be performed (3,8,19). Biopsy and direct immunofluorescence (DIF) have also been suggested (20). However, when a biopsy is performed, morphological evaluation usually reveals only nonspecific ulceration, and chronic inflammatory cell infiltrate in the lamina propria (8,20). Rarely, a biopsy is performed on an intact blister because they usually rupture easily within a few hours. In these situations, extravasation of red blood cells will be observed below the lining epithelium (8). Therefore, clinicopathological correlation is essential to ensure a correct diagnosis. The pre-sence of hemostatic disorders, antithrombotic treatment, or positive DIF may exclude the diagnosis of ABH.

Based on these observations, Ordioni *et al*. (2019) proposed nine diagnostic criteria for ABH: (I) clinically noticeable hemorrhagic blister or erosion with a history of bleeding in the oral mucosa; (II) exclusively oral or oropharyngeal localization; (III) palatal location; (IV) triggering event or promoting factor (food intake); (V) recurrent lesions; (VI) favorable evolution without leaving a scar in a few days; (VII) painless injury, or tingling or burning sensation; (VIII) normal platelet count and coagulation test; and (IX) negative DIF results (8).

These criteria were applied in all cases in our series. All patients met criteria I and II. Most patients also presented lesions predominantly located on the soft palate (n = 15, 65.2%) and favorable evolution with healing in a few days. Some also showed a triggering event or promoting factor (food intake). Direct immunofluorescence was not used as a diagnostic tool in any of the present cases. However, platelet counts and/or coagulation tests were requested in 11 patients (47.8%) to rule out hematological disorders. The results were normal. It has been suggested that a combination of at least six of the nine proposed criteria for an accurate diagnosis, with criteria I and II as required for the diagnosis of ABH (8). Although a correct diagnosis of ABH can be based exclusively on clinical criteria, additional laboratory tests may be necessary in case of persistent doubt.

Treatment of ABH is symptomatic, and the patient should be reassured (8). Analgesic drugs and local care (chlorhexidine 0.12-0.2%) can be provided. Large intact lesions, especially on the soft palate, should be incised and drained to avoid a possible obstruction of the upper aerodigestive tract (8,21). Interestingly, some authors have suggested combining ascorbic acid and citroflavonoids as a strategy to prevent recurrences (11,22).

In summary, ABH is a poorly understood disorder, and its etiology remains uncertain. Although several cases have been reported in the literature, many of them are poorly documented. Although ABH has a favorable evolution in a few days, it can share some clinical and histological characteristics with more serious diseases, making diagnosis difficult. A careful clinical examination is essential to rule out autoimmune or hematological disorders. Also, diagnostic criteria for this condition have recently been proposed based mainly on clinical examination, allowing an accurate diagnosis without the need for all complementary exams, which are sometimes unnecessary, invasive and/or costly.

## References

[1] Badham NJ (1967). Blood blisters and the oesophageal cast. J Laryngol Otol.

[2] Hopkins R, Walker DM (1985). Oral blood blisters: angina bullosa haemorrhagica. Br J Oral Maxillofac Surg.

[3] Stephenson P, Lamey PJ, Scully C, Prime SS (1987). Angina bullosa haemorrhagica: clinical and laboratory features in 30 patients. Oral Surg Oral Med Oral Pathol.

[4] Deblauwe BM, van der Waal I (1994). Blood blisters of the oral mucosa (angina bullosa haemorrhagica). J Am Acad Dermatol.

[5] Yamamoto K, Fujimoto M, Inoue M, Maeda M, Yamakawa N, Kirita T (2006). Angina bullosa hemorrhagica of the soft palate: report of 11 cases and literature review. J Oral Maxillofac Surg.

[6] Dias KB, Flores AP, Oliveira MG, Carrard VC, Hildebrand LC, Sant'Ana Filho M (2017). Angina bullosa hemorrhagica: report of 7 cases and analysis of 199 cases from the literature. Gen Dent.

[7] Alberdi-Navarro J, García-García A, Cardona-Tortajada F, Gainza-Cirauqui ML, Aguirre-Urizar JM (2020). Angina bullosa hemorrhagica, an uncommon oral disorder. Report of 4 cases. J Clin Exp Dent.

[8] Ordioni U, Hadj Saïd M, Thiery G, Campana F, Catherine JH, Lan R (2019). Angina bullosa haemorrhagica: a systematic review and proposal for diagnostic criteria. Int J Oral Maxillofac Surg.

[9] Balighi K, Daneshpazhooh M, Aghazadeh N, Rahbar Z, Mahmoudi H, Sadjadi A (2019). Angina bullosa haemorrhagica-like lesions in pemphigus vulgaris. Australas J Dermatol.

[10] Corson MA, Sloan P (1996). Angina bullosa haemorrhagica: an unusual complication following crown preparation. Br Dent J.

[11] Grinspan D, Abulafia J, Lanfranchi H (1999). Angina bullosa hemorrhagica. Int J Dermatol.

[12] High AS, Main DM (1988). Angina bullosa haemorrhagica: a complication of long-term steroid inhaler use. Br Dent J.

[13] Antoni-Bach N, Couilliet D, Garnier J, Tortel MC, Grange F, Guillaume JC (1999). Cas pour diagnostic. Stomatite bulleuse hémorragique bénigne (SBHB) [Case for diagnosis. Benign hemorrhagic bullous stomatitis]. Ann Dermatol Venereol.

[14] Higgins EM, du Vivier AW (1991). Angina bullosa haemorrhagica--a possible relation to steroid inhalers. Clin Exp Dermatol.

[15] Horie N, Kawano R, Inaba J, Numa T, Kato T, Nasu D (2008). Angina bullosa hemorrhagica of the soft palate: a clinical study of 16 cases. J Oral Sci.

[16] Edwards S, Wilkinson JD, Wojnarowska F (1990). Angina bullosa haemorrhagica--a report of three cases and review of the literature. Clin Exp Dermatol.

[17] Beguerie JR, Gonzalez S (2014). Angina bullosa hemorrhagica: report of 11 cases. Dermatol Reports.

[18] von Arx T (1998). Bullosa haemorrhagica oralis. Literaturübersicht und Fallbericht [Bullosa haemorrhagica oralis. A review of the literature and case report]. Schweiz Monatsschr Zahnmed.

[19] Scully C (2013). Recurrent oral blood blisters. J Investig Clin Dent.

[20] Giuliani M, Favia GF, Lajolo C, Miani CM (2002). Angina bullosa haemorrhagica: presentation of eight new cases and a review of the literature. Oral Dis.

[21] Pahl C, Yarrow S, Steventon N, Saeed NR, Dyar O (2004). Angina bullosa haemorrhagica presenting as acute upper airway obstruction. Br J Anaesth.

[22] Paci K, Varman KM, Sayed CJ (2016). Hemorrhagic bullae of the oral mucosa. JAAD Case Rep.

